# Elevated serum macrophage migration inhibitory factor levels correlate with benign paroxysmal positional vertigo and recurrence events

**DOI:** 10.1042/BSR20191831

**Published:** 2019-08-23

**Authors:** Hong-Bin Cai, Lei Duan, Ting Tian, Zi-Chao Li, Chong-Chong Zhao, Zhao-Ming Ge

**Affiliations:** 1 Department of Neurology, The Second Hospital of Lanzhou University, Lanzhou, China; 2 Department of Otolaryngology, The Second Hospital of Lanzhou University, Lanzhou, China

**Keywords:** Benign paroxysmal positional vertigo, inflammatory response, macrophage migration inhibitory factor, recurrence

## Abstract

**Objective:** We aimed to assess the possible relations between serum levels of macrophage migration inhibitory factor (MIF), a central cytokine of the innate immunity and inflammatory response, and benign paroxysmal positional vertigo (BPPV) risk and BPPV recurrence events.

**Methods:** In the present study, 154 patients with BPPV, and 100 age-and sex-matched control subjects were enrolled in the study. All the patients and controls underwent a complete audio‐vestibular test battery including the Dix–Hallpike maneuver and supine roll test. In the BPPV group, measurements of MIF levels were repeated 1 month after the vertigo attack. The patients were also divided into the recurrence group and the nonrecurrence group in the 1-year follow-up.

**Results**: The serum levels of MIF in patients with BPPV were higher than in those controls (13.9[interquartile range {IQR}, 8.9–18.4] ng/ml vs. 9.8[7.8–11.8]; *P*<0.001). As a continuous variable, MIF was associated with increased risk of BPPV (odds ratio [OR] 1.21, 95% confidence interval [CI]: 1.11–1.39; *P*=0.004) in multiple regression analyses. Recurrent attacks of BPPV were reported in 35 patients, and those patients had higher levels of MIF than those patients were not recurrence (18.0[IQR, 13.6–22.2] ng/ml vs. 12.6[9.3–16.8] ng/ml). In multivariate models comparing the second (Q2), third (Q3) and fourth(Q4) quartiles against the first (Q1) quartile of MIF, levels of MIF in Q4 were associated with recurrent BPPV, and the odds were increased by approximately 305% (OR = 4.05; 95%CI: 1.65–15.44; *P*=0.009).

**Conclusions**: Elevated MIF is positively correlated with BPPV risk and BPPV recurrence events, requiring further efforts to clarify the exact mechanism.

## Introduction

Of all the inner ear disorders that can cause dizziness or vertigo, benign paroxysmal positional vertigo (BPPV) is by far the most common [1]. BPPV is a disease of altered endolymph and cupular mechanics secondary to dislodged otoconia [2]. BPPV patients have episodic vertigo provoked by head movements, and they show classical horizontal, vertical or torsional nystagmus with the characteristics of latency, crescendo and decrescendo pattern, fatigability and reversibility [3]. BPPV can cause severe impact on the quality of life due to reduced daily activities, falls and depression [4]. BPPV includes two types: posterior semicircular canal (PSC) and horizontal semicircular canal (HSC). The PSC is accounting for up to 90%, whereas the HSC cases only occur in 5–15% of patients [5].

In otolaryngology, the relationship between inflammatory processes and laryngeal cancer [6], hearing loss [7], rhinosinusitis [8], otitis media [9], chronic tonsillitis [10], and inner ear disease has been reported. Macrophage migration inhibitory factor (MIF), a central cytokine of the innate immunity, is recognized as a multifunctional cytokine participating in both immune and inflammatory responses [11]. MIF might play role in the BPPV through immune and inflammatory‐mediated reactions [12]. Furthermore, a number of epidemiological studies have shown a connection between MIF levels and a wide variety of conditions, including polycystic kidney disease [13], cancer [14], stroke [15] and cardiovascular disease [16].

Some inflammatory conditions in the body, such as vasculitis, can lead to increased risk of BPPV; this can have possible effects of inflammation in inner ear disease [17]. One study suggested that interleukin 6 (IL-6) and tumor necrosis factor (TNF)-α had effective in the pathogenesis of cisplatin ototoxicity of the inner ear [18] and another study showed that IL-1β and oxidative stress contributed to the pathogenesis of BPPV [19]. Therefore, we hypothesized that MIF might be play role in the pathophysiology of BPPV. The present study thus aimed to investigate the possible of MIF, a central cytokine of the innate immunity and inflammatory response, has been proposed to play role in the development of BPPV and BPPV recurrence events in a 1-year follow-up study in Chinese patients.

## Patients and methods

This prospective one-center study was conducted from May 2017 to December 2017 at the Department of Neurology of The Second Hospital of Lanzhou University, Lanzhou, China. In the present study, one hundred and fifty-four patients with BPPV, and 100 age-and sex matched control subjects of Chinese origin ranging in age from 20 to 50 years were included. For the diagnosis of BPPV, the Dix–Hallpike maneuver was applied to patients with vestibular complaints such as vertigo, nausea, vomiting and imbalance. Worsening of the symptoms with head movements was required on history. Torsional nystagmus with latency, fatigability, and lasting shorter than 60 s in the Dix–Hallpike maneuver were considered to indicate BPPV [20].

Patients with (1) malignant; (2) liver and kidney insufficiency; (3) cardiovascular and cerebrovascular diseases or autoimmune diseases; (4) metabolic abnormalities and diabetes mellitus, hypo-or hyperthyroidism; (5) pregnancy, the use of any drug, in particular allopurinol and/or diuretics; (6) a history of antiaggregant therapy, infectious diseases or other inflammatory conditions; (7) hearing loss, a history of concomitant vestibular, otologic surgery or neurological diseases; (8) having secondary factors for BPPV, such as a history of head trauma, vestibular neuritis, Meniere’s disease, migraines, ear surgery or sudden hearing loss, having a hip or lumbar spine fracture were excluded.

For each included case, age, sex, body mass index (BMI), blood pressure (systolic blood pressure [SBP] and diastolic blood pressure [DBP]), regular physical activity habits (walking at a brisk pace for 30 min or more three times a week), smoking and drinking were recorded. BMI was calculated as weight in kilograms divided by the square of height in meters. Intensity of BPPV was assessed by the patients and expressed as visual analog scale (VAS) score (0–10), which 0 indicated no vertigo and 10 indicated severe attacks of vertigo [21].

All included subjects received a complete physical and neurotological examination. A typical history of brief attacks of positional vertigo was obtained from all patients with BPPV in whom the apparent etiology was absent and described as idiopathic [21]. Patients with BPPV underwent a complete audio-vestibular test in which eye movements were recorded by electronystagmography or videonystagmography [22]. The two types of BPPV (PSC and HSC) were diagnosed [23]. The additional characteristics of a short-latency, limited-duration intensity characterized by crescendo and decrescendo elements were also noted in conjunction with this pattern of nystagmus of intense vertigo [23]. PSC BPPV was treated by the Epley’s maneuver, whereas HSC BPPV was treated by the Barbecue maneuver, and these maneuvers had to be repeated in 10 cases [24]. The patients were also divided into the recurrence group and the nonrecurrence group. We defined recurrence of BPPV as that BPPV recurred more than one month after successful reposition, which led to the identification of nystagmus with video Frenzel glasses and consequently to the diagnosis of BPPV in the 1-year follow-up [25].

Peripheral serum samples were collected from the BPPV group at the first visit (during vertigo attack) and at the first month after the successful treatment with positioning maneuvers in patients with BPPV. Control group serum samples of healthy volunteers were collected during routine medical check-up. The samples were centrifuged for 10 min at 1500 ***g***, after which the serum was separated and stored at −80°C until further analysis. Serum levels of MIF were tested by Quantikine Human MIF Immunoassay using a commercially available enzyme linked immunosorbent assay (ELISA) kit (Catalog Number DMF00B; R&D Systems, Inc. Minneapolis, U.S.A.). According to the operating manual, the test range of the MIF was between 2 ng/ml and 100 g/ml. The coefficients of variation (CV) for the intra-and inter-assay reproducibility were all less than 8%. In addition, serum levels of C-reactive protein (CRP) and IL-6 were also tested by ELISA method. All those tested were done in duplicates and samples with a CV exceeding 10% were reanalyzed.

### Statistical analysis

Percentages for categorical variables and medians (interquartile ranges, IQRs) for continuous variables were used for expressed the results. Univariate data on demographic and clinical features were compared by Mann–Whitney *U*-test or chi-square test as appropriate. Correlations among continuous variables were assessed by the Spearman rank-correlation coefficient.

The influence of MIF levels on BPPV and recurrent BPPV were performed by binary logistic regression analysis, which allows adjustment for possible confounding factors (age, sex, BMI, SBP, DBP, smoking, drinking, VAS score, regular exercise habit, different semicircular canals and CRP/IL-6). Results were expressed as adjusted odds ratios (OR) with the corresponding 95% confidence interval (CI). In addition, multivariate analysis models were used to assess recurrent BPPV according to MIF quartiles (the lowest quartile[Q1] as the reference).

Receiver operating characteristic (ROC) curves were utilized to evaluate the accuracy of serum MIF to predict BPPV and recurrent BPPV [26]. Area under the curve (AUC) was calculated as measurements of the accuracy of the test. The cut-off value of MIF was defined according to ROC curves, and patients were divided into two groups (elevated and normal). The influence of elevated MIF levels on BPPV and recurrent BPPV were analyzed. All statistical analysis was performed with SPSS for Windows, version 22.0 (SPSS Inc., Chicago, IL, U.S.A.) and GraphPad Prism 5.0. Statistical significance was defined as *P*<0.05.

## Results

### Baseline clinical characteristics

**Figure 1:**
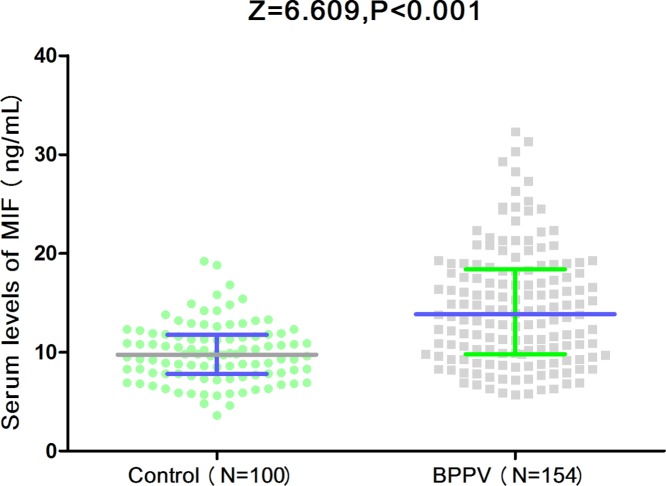
Serum levels of MIF in patients with BPPV and controls Mann–Whitney *U*-test. All data are medians and IQRs.

In the present study, 154 patients with BPPV and 100 controls were included. All the patients were evaluated within 2 weeks from the symptom onset. Clinical features and laboratory data of the patients with BPPV and control were presented in Table 1. As showed in Table 1, no significant difference of age, sex and BMI were found between the two groups (*P*>0.05). Similarly, there was also no significant association between the parameters of SBP, DBP, smoking, drinking and regular exercise habit in patients with BPPV compared with controls (*P*>0.05). The laboratory results suggested that serum levels of CRP and IL-6 reached. In addition, the median serum levels of MIF in patients with BPPV were higher than in those controls (13.9[IQR, 8.9–18.4] ng/ml vs. 9.8[7.8–11.8]; *P*<0.001) (Figure 1). The testing range of MIF in BPPV samples was from 2.2 ng/ml to 38.4 g/ml.

**Table 1:** Clinical characteristics of patients and controls

	BPPV	Controls	P^*^
*N*	154	100	
Age, median (IQR), years	37(31–43)	37(30–43)	0.84
Female, *n* (%)	85(55.2)	55(55.0)	0.98
BMI, median (IQR), kg/m^2^	25.3(24.1–27.0)	24.5(24.3–27.5)	0.18
Systolic blood pressure, median (IQR), mmHg	110(100–125)	110(95–120)	0.22
Diastolic blood pressure, median (IQR), mmHg	75(70–85)	70(66–80)	0.15
Smoking, *n* (%)	25(16.2)	16(16.0)	0.96
Drinking, *n* (%)	21(13.6)	11(11.0)	0.54
Regular exercise habit, *n* (%)	20(13.0)	12(12.0)	0.82
VAS score median (IQR)	4(2–5)	–	
Different semicircular canals, *n* (%)		–	
Posterior	120(77.9)		
Horizontal	30(19.5)		
Anterior	4(2.6)		
Laboratory testing, median (IQR)			
MIF, ng/ml	13.9(9.8–18.4)	9.8(7.8–11.8)	<0.001
IL-6, ng/ml	8.0(6.8–8.9)	7.4(5.7–8.4)	0.001
CRP, mg/l	4.3(2.9–6.7)	4.2(2.7–4.9)	0.007

* The *P* value was tested by Mann–Whitney *U*-test or Chi-Square test.

### Characteristics of BPPV

BPPV most commonly involved the PSC (77.9%), which was followed by the HSC (19.5%) and anterior canal (2.6%) (Table 1). Serum MIF levels did not differ statistically in patients with different semicircular canals (*P*>0.05). With respect to the most frequent involvement (PSC), the right side was affected in 75 patients (62.5%) and the left side in 40 patients (37.5%). MIF did not differ statistically in patients with PSC BPPV for either the right or left side (*P*>0.05). The median score of VAS was 4(IQR, 2–5). As a continuous variable, a positive correlation between VAS score and MIF was found (*r* = 0.344; *P*<0.001).

### Serum MIF levels and risk of BPPV

To identify the predictors of BPPV, univariate and multiple binary logistic regression analyses were performed. As a continuous variable, MIF was associated with increased risk of BPPV (OR 1.30, 95%CI: 1.20–1.42; *P*<0.001) in the univariate model. Multiple logistic regression analyses adjusted for age, sex, BMI, SBP, DBP, smoking, drinking, regular exercise habit, serum levels of CRP and IL-6 found that MIF was still associated with increased risk of BPPV (OR 1.21, 95%CI: 1.11–1.39; *P*=0.004).

**Figure 2:**
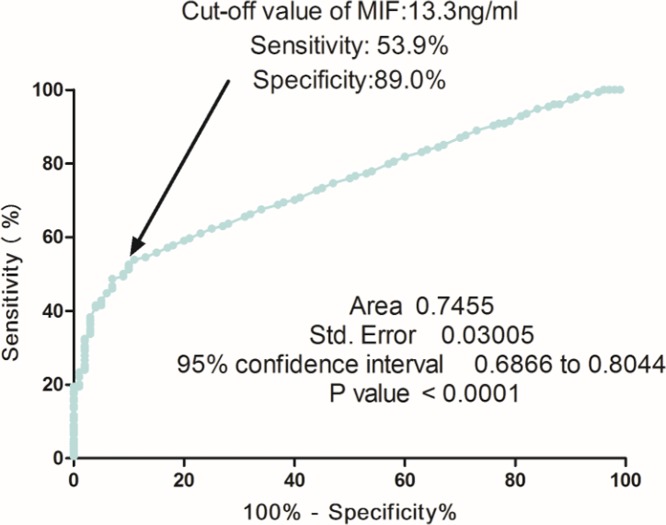
Receiver operator characteristic curve demonstrating sensitivity as a function of 1-specificity for predicting the BPPV based on serum level of MIF

Based on the ROC curve, the optimal cutoff value of serum MIF levels as an indicator for diagnosis of BPPV was projected to be 13.3 ng/ml, which yielded a sensitivity of 53.9% and a specificity of 89.0%, with the AUC at 0.75 (95%CI, 0.69–0.80) (Figure 2). Furthermore, according to this cut-off value, the patients were divided into two groups (elevated and normal). In multivariate analysis, there was an increased risk of BPPV associated with elevated MIF (OR 6.13, 95%CI: 3.15–10.16; *P*<0.001) after adjusting for above possible confounders.

### Serum MIF levels and risk of recurrent BPPV

**Figure 3:**
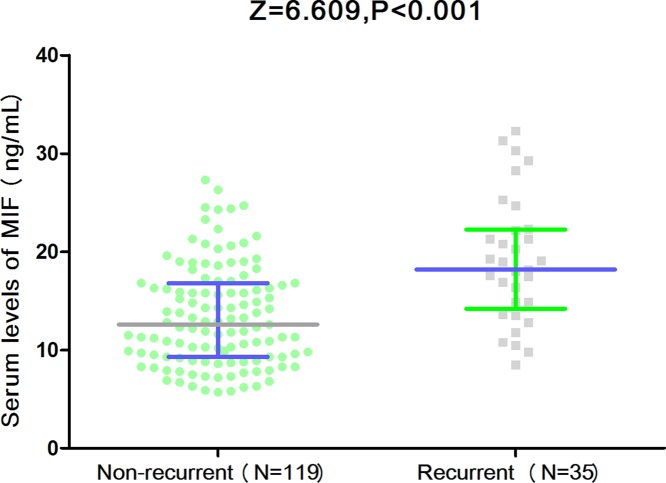
Comparisons of serum MIF levels between patients with recurrent BPPV and with non-recurrent BPPV in the1-year follow-up Mann–Whitney *U*-test. All data are medians and IQRs.

Recurrent attacks of BPPV were reported in 35 patients in the 1-year follow-up. Figure 3 showed that patients with recurrent BPPV had higher levels of MIF than those patients were not recurrence (18.0[IQR, 13.6–22.2] ng/ml vs. 12.6[9.3–16.8] ng/ml). As a continuous variable, MIF was associated with increased risk of recurrent BPPV (OR 1.17, 95%CI: 1.07–1.25; *P*<0.001) in the univariate model. Multiple logistic regression analyses adjusted for age, sex, BMI, SBP, DBP, smoking, drinking, VAS score, regular exercise habit, CRP, IL-6 and different semicircular canals showed that MIF was still associated with increased risk of recurrent BPPV (OR 1.09, 95%CI: 1.03–1.18; *P*=0.006). In addition, multivariate analysis models were used to assess recurrent BPPV according to MIF quartiles (the lowest quartile[Q1] as the reference), with the adjusted OR with 95% CIs were recorded. The recurrent BPPV rate across MIF quartiles ranged from 7.7% (first quartile) to 42.1% (fourth quartile). The *P* value for the trend was less than 0.001. In multivariate models comparing the second (Q2), third (Q3) and fourth(Q4) quartiles against the first (Q1) quartile of MIF, levels of MIF in Q4 were associated with recurrent BPPV, and the odds were increased by approximately 305% (OR = 4.05; 95%CI: 1.65–15.44; *P*=0.009). Levels of MIF in Q2 (*P*=0.52) and Q3 (*P*=0.18) did not have a statistically significance (Table 2).

**Table 2:** Univariate and multivariate analyses for recurrent BPPV according to MIF quartiles

MIF quartiles^*^	Recurrent/all	Crude OR (95%CI), *P*	Multivariable-adjusted OR^†^
Quartile 1 (Q1)	3/39	Reference	Reference
Q2	6/39	2.18(0.51–9.43), 0.29	1.48(0.42–7.89), 0.52
Q3	10/38	4.29(1.08–17.06), 0.029	3.11(0.89–11.43), 0.18
Q4	16/38	8.73(2.28–33.41), <0.001	4.05(1.65–15.44), 0.009

* MIF quartiles were defined as Q1 < 9.8 ng/ml, Q2 9.8–13.9 ng/ml, Q3 13.9–18.4 ng/ml and Q4 > 18.4 ng/ml.

† Adjusted for factors including age, sex, BMI, SBP, DBP, smoking, drinking, regular exercise habit, serum levels of CRP and IL-6.

**Figure 4:**
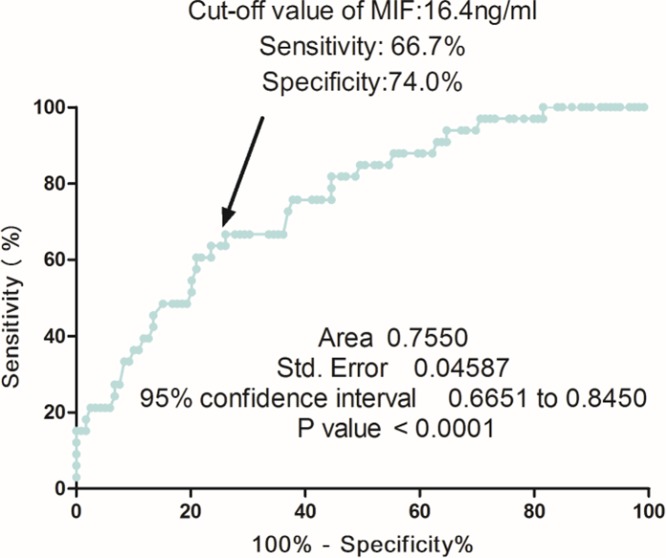
Receiver operator characteristic curve demonstrating sensitivity as a function of 1-specificity for predicting the recurrent BPPV based on serum level of MIF

Based on the ROC curve, the optimal cutoff value of serum MIF levels as an indicator for diagnosis of recurrent BPPV was projected to be 16.4 ng/ml, which yielded a sensitivity of 66.7% and a specificity of 74.0%, with the AUC at 0.76 (95%CI, 0.67–0.85) (Figure 4). Furthermore, according to this cut-off value, the patients were divided into two groups (elevated and normal). In multivariate analysis, there was an increased risk of recurrent BPPV associated with elevated MIF (OR 7.21, 95%CI: 3.77–11.72; *P*<0.001) after adjusting for above possible confounders.

### Sub-group analysis

**Figure 5:**
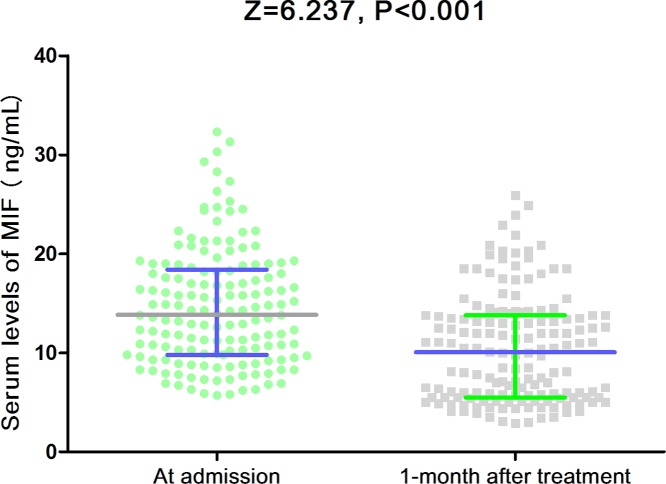
Serum levels of MIF in BPPV patients at admission and at the first month after the successful treatment Mann–Whitney *U*-test. All data are medians and IQRs.

There was a significant decrement in MIF level 1 month after the vertigo attack compared with its values during the attack (*P*<0.001; Figure 5). Furthermore, we also conducted analyses separately among men and women cases. In the multivariate regression analysis, the data showed that for each 1 ng/ml increase in serum concentration of MIF, the association with risk of BPPV was stronger among women cases (OR = 1.28, 95%CI: 1.15–1.43; *P*<0.001) versus man cases (OR = 1.18, 95%CI: 1.09–1.36; *P*=0.006). Interestingly, the predictive value of MIF to predict recurrent BPPV (OR = 1.13, 95%CI: 1.06–1.21; *P*=0.001) was also stronger in women than in men cases (1.07 [01.01–1.16]; *P*=0.009).

## Discussion

BPPV is one of the most common types of vertigo, and BPPV accounted for 8% of individuals with moderate or severe dizziness/vertigo [27]. One study reported that the lifetime prevalence of BPPV was 2.4%, the 1-year prevalence was 1.6% and the 1-year incidence was 0.6% [28]. The role of inflammatory process in BPPV has not been adequately investigated. In the present study, we evaluated serum levels of MIF in Chinese patients with idiopathic BPPV and to investigate the possible relationship between the occurrence and recurrence of BPPV and serum MIF levels. The present study showed that (1) serum levels of MIF was higher in BPPV than in controls; (2) elevated MIF was associated with the higher risk of BPPV and recurrent BPPV with an OR of 6.13 (95%CI = 3.15–10.16; *P*<0.001) and 7.21 (95%CI = 3.77–11.72; *P*<0.001), suggesting that high MIF may be a risk factor for BPPV and recurrent BPPV; (3) this correlation between MIF and BPPV was stronger in women than in men.

Inflammation and traumas in the neck or head were one of the possible etiological factors in BPPV [29]. In addition, vertigo associated anxiety may cause stress related inflammation [30]. In addition, inflammatory biomarkers from the blood samples had also be showed association with the diagnosis of BPPV [19]. Consistent with those findings, we found that MIF was associated with the diagnosis of BPPV and BPPV recurrence. However, one study found a significantly higher erythrocyte sedimentation rate (ESR) values in BPPV, while no significant differences were detected in CRP levels between patients and healthy controls [31].

The cross-sectional design prevented us from inferring any cause–effect relationship of MIF with BPPV. However, our results and previous studies indicated that MIF may be cause rather than consequence of BPPV recurrence events. The inner ear is a unique organ with a blood-labyrinthine barrier like the brain, has connections with cervical lymph nodes, and is capable of cytokine production in the spiral ligament; hence, with cervical lymph node connections, the inner ear is under the control of systemic T-lymphocytes for immune responses [32]. Rheumatoid arthritis, an immune-mediated systemic disorder, can also affect the inner ear and can cause high-frequency hearing loss [33]. An inflammatory process maybe initiated by the inner ear and it can result in hearing loss. It has been shown that cisplatin induced ototoxicity could be related with some inflammatory mediators such as IL-1, IL-6 and TNF-α [18]. Previous studies revealed that systemic sclerosis and giant cell arteritis patients experienced a higher percentage of BPPV, and the role of immune-mediated inflammatory process has been reported [19,34]. A hypothetical mechanism of inflammation in the pathogenesis of BPPV was reported in another study. They revealed a strong co-existence between stress and BPPV, and reported, that stress-related inflammation maybe a possible mechanism in the pathogenesis of BPPV [35].

Furthermore, previous studies investigated the direct role of oxidative stress in BPPV. Goto et al. [36] studied the probable role of angiitis and diacron reactive oxygen metabolites (d-ROM) in BPPV. Tsai et al. [37] found that the serum levels of the oxidative stress marker malondialdehyde were higher in the BPPV group before the repositioning maneuver. Another study suggested a role of oxidative stress in the development of BPPV, through both calcium metabolism and the direct toxic effects of free oxygen radicals, including the triggering of apoptosis [38]. Interestingly, Nguyen et al. [39] suggested that MIF was a modulator of pro-oxidative stress-induced apoptosis. Furthermore, reactive oxygen species (ROS) and oxidized lipoproteins play role in the activation of inflammatory cells and inducing signaling pathways related to cell death and apoptosis [40]. Lastly, calcium and carbonate levels in the endolymph are critical for normal otoconial function, which is primarily provided by a calcium channel transport system expressed in the inner ear [41]. High-grade disintegration in the otoconia caused complete dissolution and fragmentation of calcium-rich material to the endolymph, creating the possible underlying cause of symptoms in BPPV [19]. An inflammation-induced increase in evoked Ca^2+^ transients in putative nociceptive afferents may contribute to the pain and hyperalgesia associated with persistent inflammation via facilitation of transmitter release from these afferents [42].

Some limitations should be considered. First, this study was lack of functional assessment of HPA axis in parallel and no information about MIF gene expression. Second, despite many recently reported neurotological research studies, currently we do not have access to the inner ear in order to uncover the actual chemistry of the endolymph in real time, which represents the greatest limitation of these studies. In addition, despite the fact that the groups were identical for age and gender, it is clearly unrealistic to homogenize all environmental and demographic factors for all enrolled subjects. Third, it was a single-center study and the number of patients were relatively low (*N* = 154). Lastly, observational study design did not allow drawing secure conclusions for a causal relationship. It was unclear whether the biomarkers and systemic inflammation had a cause and effect relationship in our study, and further large-scale basic and clinical studies are required to confirm this relationship.

## Conclusions

The present study showed that serum levels of MIF was higher in BPPV than in controls, suggesting a role of MIF in the risk of BPPV. Elevated serum levels of MIF were associated with increased risk of recurrent BPPV in the next 1 year and might be useful in identifying BPPV at risk for recurrence events for early prevention strategies. Further studies are proposed to confirm this association, which may open a good drug target for the therapy of BPPV.

## Availability of data and material

Please contact the correspondence author for the data request.

## Abbreviations

AUC: area under the curve
BMI: body mass index
BPPV: benign paroxysmal positional vertigo
CI: confidence interval
CRP: C-reactive protein
CV: coefficients of variation
DBP: diastolic blood pressure
HSC: horizontal semicircular canal
IL-6: interleukin 6
IQR: interquartile range
MIF: macrophage migration inhibitory factor
OR: odds ratio
PSC: posterior semicircular canal
ROC: receiver operating characteristic
SBP: systolic blood pressure
TNF: tumor necrosis factor
VAS: visual analog scale
